# Crowdsourcing Adverse Events Associated With Monoclonal Antibodies Targeting Calcitonin Gene–Related Peptide Signaling for Migraine Prevention: Natural Language Processing Analysis of Social Media

**DOI:** 10.2196/58176

**Published:** 2024-11-08

**Authors:** Pengfei Zhang, Brad K Kamitaki, Thien Phu Do

**Affiliations:** 1 Department of Neurology Rutgers-Robert Wood Johnson Medical School New Brunswick, NJ United States; 2 Department of Neurology Danish Headache Center Copenhagen University Hospital - Rigshospitalet Copenhagen Denmark; 3 Department of Neurology Copenhagen University Hospital - Herlev and Gentofte Herlev Denmark; 4 Danish Knowledge Center on Headache Disorders Glostrup Denmark; 5 Department of Clinical Medicine University of Copenhagen Copenhagen Denmark

**Keywords:** internet, patient reported outcome, headache, health information, Reddit, registry, monoclonal antibody, crowdsourcing, postmarketing, safety, surveillance, migraine, preventives, prevention, self-reported, calcitonin gene–related peptide, calcitonin, therapeutics, social media, medication-related, posts, propranolol, topiramate, erenumab, fremanezumab, cross-sectional, surveys

## Abstract

**Background:**

Clinical trials demonstrate the efficacy and tolerability of medications targeting calcitonin gene–related peptide (CGRP) signaling for migraine prevention. However, these trials may not accurately reflect the real-world experiences of more diverse and heterogeneous patient populations, who often have higher disease burden and more comorbidities. Therefore, postmarketing safety surveillance is warranted. Regulatory organizations encourage marketing authorization holders to screen digital media for suspected adverse reactions, applying the same requirements as for spontaneous reports. Real-world data from social media platforms constitute a potential venue to capture diverse patient experiences and help detect treatment-related adverse events. However, while social media holds promise for this purpose, its use in pharmacovigilance is still in its early stages. Computational linguistics, which involves the automatic manipulation and quantitative analysis of oral or written language, offers a potential method for exploring this content.

**Objective:**

This study aims to characterize adverse events related to monoclonal antibodies targeting CGRP signaling on Reddit, a large online social media forum, by using computational linguistics.

**Methods:**

We examined differences in word frequencies from medication-related posts on the Reddit subforum r/Migraine over a 10-year period (2010-2020) using computational linguistics. The study had 2 phases: a validation phase and an application phase. In the validation phase, we compared posts about propranolol and topiramate, as well as posts about each medication against randomly selected posts, to identify known and expected adverse events. In the application phase, we analyzed posts discussing 2 monoclonal antibodies targeting CGRP signaling—erenumab and fremanezumab—to identify potential adverse events for these medications.

**Results:**

From 22,467 Reddit r/Migraine posts, we extracted 402 (2%) propranolol posts, 1423 (6.33%) topiramate posts, 468 (2.08%) erenumab posts, and 73 (0.32%) fremanezumab posts. Comparing topiramate against propranolol identified several expected adverse events, for example, “appetite,” “weight,” “taste,” “foggy,” “forgetful,” and “dizziness.” Comparing erenumab against a random selection of terms identified “constipation” as a recurring keyword. Comparing erenumab against fremanezumab identified “constipation,” “depression,” “vomiting,” and “muscle” as keywords. No adverse events were identified for fremanezumab.

**Conclusions:**

The validation phase of our study accurately identified common adverse events for oral migraine preventive medications. For example, typical adverse events such as “appetite” and “dizziness” were mentioned in posts about topiramate. When we applied this methodology to monoclonal antibodies targeting CGRP or its receptor—fremanezumab and erenumab, respectively—we found no definite adverse events for fremanezumab. However, notable flagged words for erenumab included “constipation,” “depression,” and “vomiting.” In conclusion, computational linguistics applied to social media may help identify potential adverse events for novel therapeutics. While social media data show promise for pharmacovigilance, further work is needed to improve its reliability and usability.

## Introduction

Preventive medications for migraine include both non–disease-specific drugs (eg, antihypertensives and anticonvulsants) and, more recently, disease-specific anti–calcitonin gene–related peptide (CGRP) therapies [1]. Randomized controlled trials (RCTs) demonstrate the clinically relevant efficacy and tolerability of anti-CGRP monoclonal antibodies (mAbs) [2]. However, they may not accurately reflect the real-world experiences of more heterogeneous and diverse patient populations who often have a higher disease burden and more comorbidities [3-6]. In addition to direct reports of potential adverse events from health care providers and patients, regulatory organizations encourage marketing authorization holders to screen digital media for suspected adverse reactions, which should be handled similarly to spontaneous reports with the same requirements applied [7]. While social media represents an obvious venue in this context, its usage for pharmacovigilance is still in its infancy [8]. Complementary methods of postmarketing surveillance for adverse events with social media are needed.

The last 15 years were characterized by a vast proliferation of social media platforms, allowing for both the rapid dissemination of medical information, as well as direct insight into patient perspectives. Indeed, migraine is most prevalent in individuals between 15-49 years of age, who are among the most active users of the internet [9]. This is also reflected by the growth of migraine-related content on the internet [10-12]. The anonymous social media discussion board Reddit has over 430 million monthly active users and serves as a popular forum for health-related discussions [13]. Unlike other digital media platforms, Reddit provides easily accessible and legal ways of accessing historic forum data. Anonymous forums like Reddit allow users to share personal medical experiences and advice without social stigma. Collectively, the migraine-related subforums (or “subreddits”) such as r/Migraine have a user base exceeding 54,100. While these platforms allow for a democratic exploration of patient experiences and perspectives, they are relatively understudied.

Computational linguistics, a discipline encompassing automatic manipulation and quantitative analysis of oral or written language, represents a potential way to explore this content. Computational linguistics can be applied to identify clusters of words in a dataset with the goal of deriving dominant thematic elements. Identifying word distribution frequencies in specific groups of texts is a common technique used in computational linguistics with multiple wide-ranging applications [14]. In this study, we applied computational linguistics, specifically word frequency analysis, to characterize and identify self-reported adverse events associated with the new therapeutic anti-CGRP mAbs reported by users on Reddit. To ascertain the reliability and validity of our methodology, we first conducted a preliminary study with older-generation migraine drugs to see if we could identify known adverse reactions. After validation, we applied the methodology to the newer generation of CGRP therapies for migraine prevention.

## Methods

### Study Design

This study was a cross-sectional survey of posts on the r/Migraine subforum on Reddit. Data were analyzed using custom back-end software. The study consisted of four phases: (1) data extraction, (2) automated data organization, (3) validation of computational linguistics approach, and (4) application of computational linguistics approach.

#### Data Extraction

We extracted posts in JavaScript Object Notation format from the Reddit r/Migraine subforum published from January 1, 2010, to January 1, 2020, using Pushshift, a publicly available Reddit repository, in order to extract data for analysis [15]. The time stamp, title, and text of each post were obtained and combined into a comma-separated values file for further evaluation. Of note, our search did not include any comments responding to individual posts. Furthermore, usernames were excluded to ensure the protection of personal data. Once the above data were downloaded, we then converted the entire document to lowercase characters for ease of data search and extraction.

#### Automated Data Organization

For the validation phase of the study, we algorithmically obtained, through the Haskell filter function, a list of documents containing posts with either the brand or trade name for propranolol and topiramate. These medications were selected as evidence-based preventive treatments for migraine in accordance with American Headache Society guidelines and because their side effect profiles are well known [16]. Next, we algorithmically compiled all posts containing each drug in separate documents. To use computational linguistics terminology, each document represents a corpus for a medication; each post within that corpus further represents a text.

We subsequently identified posts not including any of the above medications, nor including any CGRP therapeutic medication, to serve as controls for comparison. To conduct multiple tests, we randomly divided these controls into 4 groups: group 1, group 2, group 3, and group 4. To accomplish this, we assigned each post in the control a random number between 1 to 4. All posts with randomly assigned digits 1 were grouped into group 1. Those with a digit of 2 were grouped into group 2, and so on.

#### Validation of Computational Linguistics Approach

Comparing the relative occurrence of words between 2 groups of texts is a well-defined technique in computational linguistics. For drugs with different distinct side effect profiles (eg, the anticonvulsant topiramate vs the beta-blocker propranolol), we hypothesized there would be a difference in the frequency of word distributions between corpora for these different medications.

Although the chi-square test or log-likelihood ratio test are commonly used, Lijiffijit et al [17] recently demonstrated that the Wilcoxon rank-sum test, Welch *t* test, or bootstrap test are better choices to use to avoid overconfidence bias. Consequently, we used and validated Welch *t* test for each of these comparisons given the ease of implementation and intuitiveness.

In accordance with the abovementioned methodology, given a corpus for drug S and a corpus for drug T, we denoted each individual word by q, the total number of words in each corpus by n_1_ and n_2_, and x_1_ and x_2_ as the mean frequency of q over corpus S and corpus T, respectively. We then applied Welch *t* test [17]. To simplify our calculations, words with fewer than 3 characters in length, symbols, and numbers were filtered out. We identified any words showing statistical significance at *P*<.05 using a 1-tailed *t* test since to reject the null hypothesis, it is sufficient to show that a specific word occurrence is greater—not less—in 1 corpus against another, or a *t* statistic>1.73. Because this process involved the repeated application of Welch *t* test n_1_+ n_2_ times to the same dataset, the Benjamini-Hochberg procedure was used to decrease multisampling bias. Specifically, once statistically significant words were obtained after a given comparison between 2 corpora, the list of significant words was then ranked by *P* value. We then applied the Benjamini-Hochberg procedure to this ranked ordered list, only retaining those words passing this procedure.

To validate our method and the reliability of the Reddit data, we applied this approach first by comparing propranolol and topiramate. Of note, if a text existed in both the propranolol and the topiramate corpora, then it was taken out of both. Subsequently, we compared propranolol versus group 1 and topiramate versus group 2.

#### Application of Computational Linguistics Approach

The above approach was then applied to the corpus between erenumab versus group 3 in order to establish a baseline side effect profile for erenumab. Similarly, fremanezumab was compared to group 4 to establish a baseline side effect profile for fremanezumab. We then compared the erenumab corpus against the fremanezumab corpus to ascertain any difference between the 2 medication side effect profiles. This is clinically relevant, as erenumab targets the CGRP receptor, while fremanezumab targets the CGRP ligand. We were interested to see whether there were fewer reports of specific adverse events with erenumab than with fremanezumab. Again, when comparing erenumab and fremanezumab, shared posts were removed.

For evaluation of our data, all contributing authors reviewed words identified through the above processes, where a word is counted as describing a potential adverse event by consensus. The above algorithms, including data download, parsing, filtering, and data analysis were implemented through custom back-end software using a combination of the Clojure and Haskell programming languages.

### Ethical Considerations

Our data source was obtained from Pushshift, a social media data collection site, with the explicit purpose of collecting Reddit data for the purpose of research [15]. Our project proposal was approved by the Rutgers University institutional review board as nonhuman subjects research (Pro2020001648). To ensure the anonymity of users in our data processing, we removed all usernames from our dataset. Furthermore, since our methodology examines posts in aggregate, individual identifiable data were not published nor evaluated as part of this study.

## Results

### Dataset

**Figure 1:**
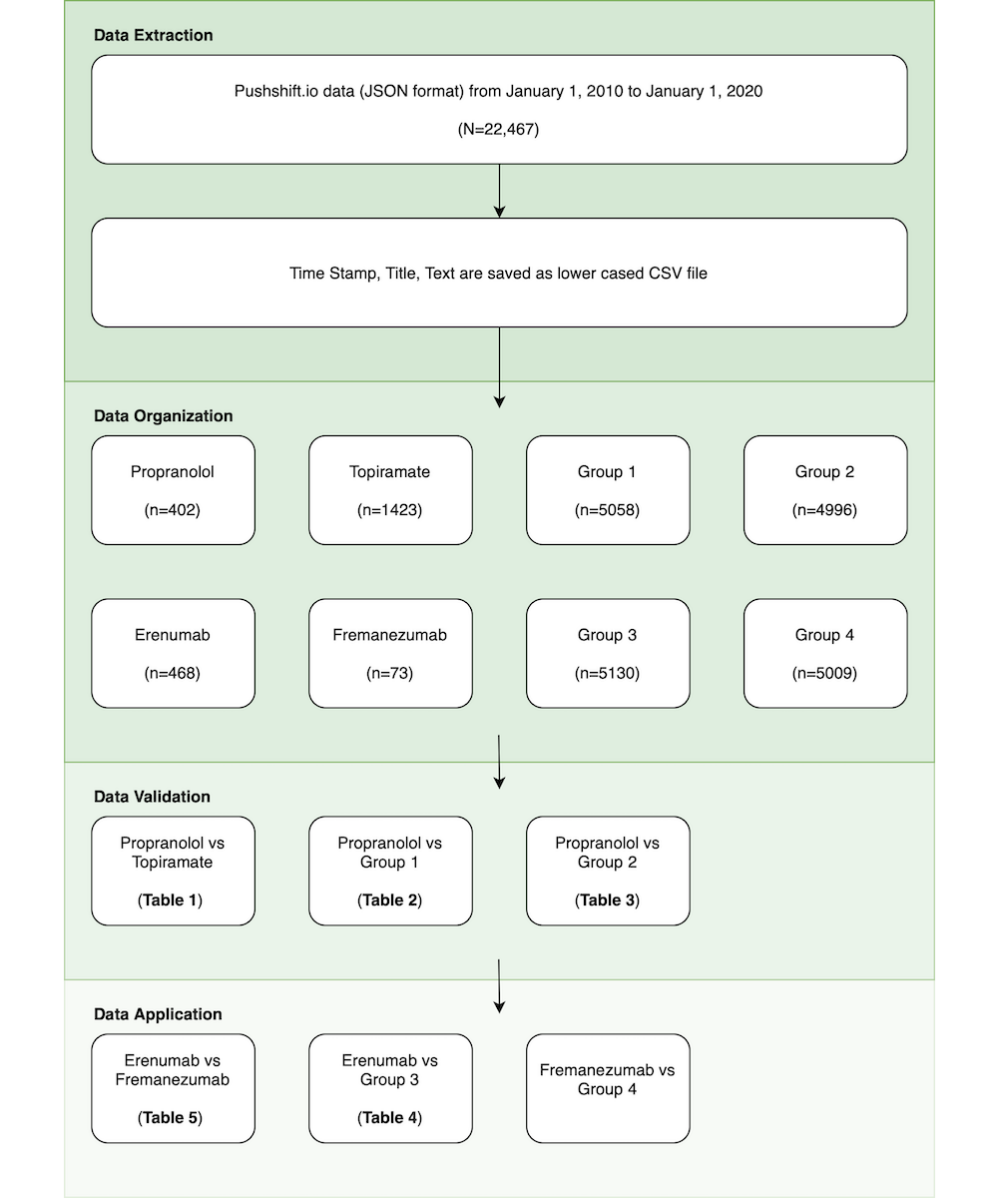
Study flow diagram of the validation and application phases of a computer linguistics analysis of r/Migraine social media posts on Reddit.

We extracted a total of 22,467 Reddit posts from r/Migraine, spanning from January 1, 2010, to December 31, 2019. In the validation phase of the study, we identified 402 (2%) posts that mentioned propranolol and 1423 (6.33%) posts that mentioned topiramate. During the application phase, focusing on mAbs targeting CGRP signaling, we found 468 (2.08%) posts that mentioned erenumab and 73 (0.32%) posts that mentioned fremanezumab (Figure 1).

Since these posts did not exclusively reference the target medications, additional steps were taken to exclude co-occurring texts when comparing medications, as detailed in the *Methods* section. For the control corpora, group 1 contained 5058 posts, group 2 had 4996 posts, group 3 included 5130 posts, and group 4 comprised 5009 posts.

### Validation of Computational Linguistics Approach

When topiramate posts were compared against propranolol posts using the earlier methodology, side effect–related words including “appetite,” “cognitive,” “tingling,” “remember,” “taste,” “dopamax” (a frequently used slang term describing the cognitive adverse events of topiramate), “tastes,” and “kidney” were statistically more frequent (Textbox 1).

**Keywords identified when comparing topiramate with propranolol from the Reddit r/Migraine subforum (2010-2020). Italicized words were identified as potential side effects–related words of interest.**

topamax, topiramate, *appetite*, effects, posts, ready, realized, serious, throw, *cognitive*, loss, side, treating, level, flat, *tingling*, situation, blocks, opinion, improved, ntopamax, options, woman, possibility, issues, reddit, glasses, awesome, cymbalta, grateful, *remember*, version, depressants, decent, dumb, believe, future, *taste*, angry, evidence, *dopamax*, tapering, someone, practice, trust, shortly, honest, sign, *kidney*, steroids, refused, tons, disorders, cambia, learned, nerve, feet, ahead, essentially, posted, *tastes*, allergic, emotional, harder, ride, limited, white, spoke, parts, comment, optimistic, hurt, count

When propranolol was compared to the control corpus group 1, a total of 98 words appeared more frequently, none of which we identified as potential adverse events (Textbox 2). When topiramate was compared against group 2, there were 563 words that appeared more often (Textbox 3). The following words were determined to be potential adverse effects: “appetite,” “weight,” “remember,” “memory,” “cognitive,” “anxiety,” “taste,” “zombie,” “tingly,” “dizzy,” “fatigue,” “forget,” “kidney,” “foggy,” “dopamax,” “forgetful,” “dizziness,” “carbonate,” and “tastes.”

**Keywords identified when comparing propranolol with group 1 from the Reddit r/Migraine subforum (2010-2020).**

propranolol, taking, been, months, topamax, effects, prescribed, tried, with, take, medication, doctor, side, dose, have, neurologist, preventative, amitriptyline, daily, since, also, inderal, currently, anyone, started, month, years, weeks, from, beta, which, migraines, medications, some, about, life, week, meds, made, drug, really, time, year, days, will, other, first, nortriptyline, after, because, dosage, effect, again, just, didn, stopped, they, haven, topiramate, help, well, terrible, went, starting, this, past, blockers, still, doesn, almost, maxalt, nothing, feel, sumatriptan, that, back, like, seemed, what, stop, preventatives, appointment, long, good, advice, weight, blood, npropranolol, give, neuro, blocker, relief, imitrex, worked, results, going, headaches, only

**Keywords identified when comparing topiramate with group 2 from the Reddit r/Migraine subforum (2010-2020). Italicized words were identified as potential side effect–related words of interest.**

topamax, side, effects, taking, topiramate, been, have, with, neurologist, just, dose, about, that, take, started, tried, week, this, since, which, months, doctor, because, month, migraines, weeks, from, also, some, well, daily, other, prescribed, like, years, going, back, what, effect, really, preventative, only, work, working, neuro, days, made, know, much, long, loss, starting, need, even, after, currently, first, medication, meds, still, worked, didn, time, then, propranolol, again, good, stopped, taken, more, nothing, point, *appetite*, dosage, drug, imitrex, would, helped, when, control, works, medications, getting, night, will, think, preventatives, already, were, there, every, little, *weight*, went, amitriptyline, gave, said, issues, appointment, maxalt, increased, want, last, should, feel, anything, anyone, trying, seems, being, before, pretty, very, having, wants, here, finally, twice, until, able, give, helping, *remember*, while, chronic, where, experiences, haven, experience, next, over, three, year, triptans, stop, used, options, down, botox, could, severe, into, better, keep, they, beta, away, help, read, worse, blockers, birth, through, great, them, feet, *memory*, success, depakote, though, another, almost, increase, past, doses, under, female, anymore, these, frequency, decided, seeing, same, many, effective, switch, came, symptoms, actually, gabapentin, magnesium, different, without, needed, aimovig, else, seem, depression, lost, course, things, recently, thing, half, trokendi, lose, advice, desperate, wanted, problems, increasing, horrible, switched, diagnosed, couldn, took, normal, life, *tingling*, history, start, suggested, mood, drugs, several, sure, doesn, maybe, concerned, awful, *cognitive*, constant, might, find, *anxiety*, insurance, wasn, changed, called, experiencing, break, everything, feeling, doing, agreed, medicine, reading, function, never, makes, drive, neurologists, abortive, nthanks, talk, times, sent, headaches, ntopamax, myself, anti, nortriptyline, pill, treatment, verapamil, prescription, instead, results, than, told, once, withdrawal, prevention, nervous, supposed, august, doctors, seen, relief, probably, including, less, completely, worth, hands, felt, switching, nausea, finding, primary, care, reaction, pills, either, hoping, tired, change, least, antidepressants, continue, prescribe, april, something, plus, fioricet, background, post, period, current, basically, those, hell, deal, frustrated, hello, difference, question, sleepy, afraid, however, story, *taste*, around, cannot, list, terrible, says, cymbalta, wondering, stopping, suggestions, idea, medical, asked, *zombie*, fine, issue, such, serious, hard, combo, realize, right, changes, july, blood, body, pharmacist, initially, brain, ready, thinks, treating, happy, tonight, gone, suffering, people, done, developed, talked, *tingly*, abortives, supplements, couple, risk, small, frequent, stories, flat, became, eating, looking, zomig, stuff, free, gained, problem, stayed, *dizzy*, *fatigue*, opinion, wean, except, given, hair, gain, situation, possible, lasted, various, pounds, zero, words, causing, minimal, metoprolol, *forget*, reduced, taper, steroids, physician, relpax, definitely, single, upped, *kidney*, ntopiramate, possibly, eventually, weaning, steroid, elavil, tests, making, nafter, whole, exercise, insomnia, january, ever, zonegran, september, pain, thinking, second, generic, slowly, depressants, follow, major, *foggy*, *dopamax*, please, specialist, nsaids, level, functional, plan, monday, hospital, shot, drink, kept, reduction, toradol, none, surprised, gotten, cycle, rescue, positive, seemed, intractable, stay, make, failed, chance, dropped, ncurrently, nightly, crazy, live, acupuncture, average, family, seizure, *forgetful*, levels, quit, stomach, calcium, ability, early, practice, selftext, others, thanks, discuss, occasionally, june, propanolol, high, most, decrease, schedule, losing, gradually, believe, guess, both, stones, coming, bring, march, everyone, look, biggest, previous, option, higher, helps, found, longer, tolerance, october, onto, slow, morning, previously, constantly, wits, fairly, negative, especially, tapering, proven, posts, quitting, upping, common, kind, reason, possibility, number, waiting, online, bedtime, goes, name, pharmacy, best, super, improved, handle, treat, nbut, *dizziness*, using, thank, complete, main, vitamins, fast, stick, anyway, bumped, willing, miracle, hasn, shocked, clinic, decent, mris, noticed, weaned, refused, talking, candidate, together, quick, nanyway, occasional, round, efficacy, tolerate, improvement, *carbonated*, nany, suggest, chest, *tastes*, psychiatrist

### Application of Computational Linguistics Approach

We next identified frequent words of interest by comparing erenumab to group 3 (Textbox 4) and fremanezumab to group 4. For erenumab, 36 words appeared more frequently, but “constipation” was the only potential adverse effect. The only word appearing more often for fremanezumab versus the control corpus was “Ajovy,” a brand name for fremanezumab.

**Keywords identified when comparing erenumab with group 3 from the Reddit r/Migraine subforum (2010-2020). Italicized words were identified as potential side effects–related words of interest.**

aimovig, dose, month, injection, botox, neurologist, effects, months, drug, first, insurance, emgality, injections, tried, topamax, side, free, trial, neuro, next, shot, approved, preventatives, since, hoping, ajovy, cgrp, *constipation*, effect, plan, working, here, received, shots, doses, propranolol

When comparing erenumab to fremanezumab, there were 180 words that were significantly associated with erenumab, with the following words representing potential adverse events: “depression,” “constipation,” and “vomiting” (Textbox 5). Notably, the word “muscle” was also associated with erenumab, but it was unclear whether this described an adverse event (Textbox 5). The only word considered significant for fremanezumab was “Ajovy” with no adverse effect–related words detected.

**Keywords identified when comparing erenumab with fremanezumab from the Reddit r/Migraine subforum (2010-2020). Italicized words were identified as potential side effect–related words of interest.**

aimovig, where, felt, medications, doesn, effect, much, starting, makes, enough, stopped, such, come, everything, home, triptan, next, possible, half, wait, worth, being, live, high, talk, preventatives, trial, question, nhas, medicine, diagnosed, thinking, hour, means, received, know, definitely, best, caused, into, happy, sumatriptan, given, lost, issue, short, here, *depression*, frustrated, early, should, weird, aren, knows, problem, especially, leave, advance, mind, ones, painful, story, *constipation*, real, cost, school, list, thank, related, note, support, told, nothing, this, follow, nbut, nthis, stories, wouldn, another, available, https, thread, difficult, info, okay, regular, bring, vision, wrong, migraine, specialist, often, made, abortive, considering, history, intense, notice, sometimes, sort, *vomiting*, break, causing, expensive, experiencing, learned, middle, asking, physical, seemed, vent, muscle, coming, concerned, monday, water, current, program, work, changes, medical, make, didn, blocks, happen, recommended, along, case, horrible, extra, specific, chest, fact, fall, letting, nalso, reason, types, type, serious, reading, barely, situation, therapy, treatments, erenumab, nand, began, clinic, dealing, minute, trials, freaking, ideas, tuesday, incredibly, move, nfor, paying, strong, benefits, continue, friends, mostly, naimovig, ndoes, news, nnow, rest, unfortunately, wonder, hold, reddit, update, toradol, prescribe, disease, switched, rebound

## Discussion

In this study, we applied computational linguistics methods to unfiltered discussions about migraine treatments on Reddit to distinguish patient-reported adverse effects associated with these medications. The validation phase of our study accurately identified common adverse events for standard migraine preventive treatments. For example, typical adverse events such as “taste” and “tingling” appeared in posts for topiramate. After applying this methodology to mAbs targeting CGRP or its receptor—fremanezumab and erenumab, respectively—no definite adverse events were flagged for fremanezumab. Notable flagged words for erenumab included “constipation,” “depression,” and “vomiting.”

In our proof-of-concept analysis, we confirmed that keywords discussed in relation to topiramate from the Reddit r/Migraine subforum are consistent with the most common adverse events observed in RCTs [18]. Interestingly, dizziness, a common side effect, did not appear when comparing propranolol to either the control corpus or topiramate [19]. A possible explanation could be that many different words were used to describe this side effect, for example, “lightheadedness” and “dizzy,” which were detected individually but did not meet statistical significance after correcting for multiple comparisons. Because patients may use a variety of different words to describe a single symptom, pooling these terms be useful in future studies when analyzing social media data.

While constipation was not a major concern during RCTs with erenumab, emerging real-world data report that it is among the most frequent reasons for treatment discontinuation [20,21]. Constipation was subsequently added to the US Food and Drug Administration (FDA) label after regulatory approval [22]. During RCTs, approximately 2% of participants reported constipation compared to approximately 10% to 20% of real-world users [3,20,23]. Our findings further support this, since Reddit users frequently discuss constipation and vomiting in relation to erenumab. The discrepancy between data from RCTs and real-world experiences may be due to a more heterogeneous patient population receiving erenumab in the postmarketing phase. This population likely includes patients with other medical comorbidities or factors that could have excluded them from consideration in clinical trials. Increasing evidence suggests that individuals with migraine may have a higher prevalence of comorbid gastrointestinal disorders when compared with the general population [24,25].

Interestingly, the word “muscle” was associated with posts discussing erenumab when compared with fremanezumab. Although we did not include this word as a potential adverse event due to its ambiguity, it could be related to muscle cramps, which were added to the FDA warning label for erenumab after approval [22]. Muscle spasms occurred in approximately 2% of patients in RCTs [20]. Whether “muscle”-related adverse effects are occurring at a more significant rate in real-world use is unclear. Additional investigation into the extent and severity of muscle disease due to erenumab could be considered in future studies or when examining other postmarketing surveillance data sources for verification.

Another notable postmarketing safety update was the addition of hypertension to the FDA label for erenumab; however, this was not flagged in our analysis. In RCTs, less than 1% of participants reported hypertension, but participants with severe cardiovascular risk factors were excluded from these trials, and hypertension has not been commonly noted in other studies [3,23]. It is possible that cardiovascular risk factors are screened to a higher degree than gastrointestinal disorders. Patients at risk for hypertensive complications might, therefore, be more likely to decline treatment with this drug. Finally, worsening of hypertension is typically asymptomatic, and patients may not recognize this as a potential adverse event to raise in social media discussions.

There were no keywords flagged in the fremanezumab dataset. The finding that constipation was flagged for erenumab but not for fremanezumab could be related to pharmacodynamic differences in the mechanism of action. Erenumab targets the CGRP receptor, while fremanezumab targets its ligand. One study offers a possible mechanistic model that strengthens this hypothesis, specifically, via direct effects of CGRP on intestinal motility modulated by other receptors in addition to the canonical CGRP receptor [26]. However, galcanezumab, another mAb targeting the CGRP ligand, also had constipation listed as a common adverse reaction in clinical studies and postmarketing reports [27]. Further data are needed to clarify any potential differences in mechanism.

A strength of our study is the inherent anonymity of the Reddit forum, which allows us to analyze information that patients might not otherwise feel comfortable discussing or prioritize sharing during a clinical consultation. Querying the Reddit migraine subforum also provides insight into the experience of more than 54,000 unique users, which could include diverse sources ranging from patients and family members to experienced health care providers.

However, the use of Reddit data also confers significant limitations. We cannot correlate our findings with individual patient demographics, previous medical history, or other clinically relevant information. All information is self-reported and subjective, which calls into question the accuracy of unverifiable data. Although the number of posts differs between erenumab and fremanezumab due to FDA approval time, we do not expect this to significantly change our result since word distribution should be theoretically constant despite the sample size. Another limitation is the use of multiple different words for the same symptom; for example, when “lightheadedness,” “dizzy,” and “dizziness” collectively describe the same entity. Relevant symptoms described in various ways may be missed during this type of analysis. Pooling multiple similar terms could avoid this problem in future studies. Finally, our method did not analyze comments responding to specific posts; incorporating this into future studies using word frequency methodology could increase the accuracy of our conclusions. Future directions for our line of research include the analysis of other digital platforms to compare with our findings.

Postmarketing pharmacovigilance remains critical, especially when RCTs underdetect adverse events. When compared with health care professionals, direct patient reporting of adverse drug events identifies more potential signals and in greater detail [28]. Our study provides support that monitoring patient reports via social media can be incorporated into postmarketing safety surveillance for preventive migraine medications. While our proposed methodology has its limitations, the data offer a broader postmarketing perspective that complements clinical trial findings and case reports. We hope that consideration by relevant stakeholders will enhance existing tools in the pharmacovigilance armamentarium, enabling quicker and earlier detection of adverse effect signals. However, more work is needed to improve the reliability and validity of social media analysis, which faces the same challenges inherent to big data, including high volume, unstructured capture, and structural regulatory barriers, which all need to be addressed before implementation [29].

## Abbreviations

CGRP: calcitonin gene–related peptide
FDA: Food and Drug Administration
mAb: monoclonal antibody
RCT: randomized controlled trial

## Data Availability

The datasets generated during and/or analyzed during this study are available from the corresponding author on reasonable request.
